# Enrollment and Completion Characteristics for Novel Remote Delivery Modes of the Self-management Programs During the COVID-19 Pandemic: Exploratory Analysis

**DOI:** 10.2196/38357

**Published:** 2022-11-30

**Authors:** Kristin Pullyblank, Serdar Atav

**Affiliations:** 1 Center for Rural Community Health Bassett Research Institute Bassett Medical Center Cooperstown, NY United States; 2 Decker College of Nursing and Health Sciences Binghamton University State University of New York Binghamton, NY United States

**Keywords:** self-management programs, self management, internet-based intervention, health promotion, COVID-19, health equity, socioeconomic status, remote healthcare, health delivery, virtual care, remote care, remote delivery, videoconference, videoconferencing, adherence, attrition, completion, virtual health

## Abstract

**Background:**

In-person, evidence-based, peer-facilitated chronic disease self-management programs have been shown to be effective for individuals from a variety of backgrounds, including rural and minority populations and those with lower socioeconomic status. Based in social learning theory, these programs use group processes to help participants better manage their chronic disease symptoms and improve their quality of life. During the pandemic, these in-person programs were forced to rapidly transition to remote delivery platforms, and it was unclear whether doing so increased disparities within our rural population.

**Objective:**

The objectives of this analysis were to ascertain self-management program enrollment and completion characteristics between 2 remote delivery platforms, as well as determine the individual level characteristics that drove enrollment and completion across delivery modes.

**Methods:**

We analyzed enrollment and completion characteristics of 183 individuals who either enrolled in a self-management workshop delivered through a web-based videoconference (VC) system or through a traditional, audio-only conference call (CC) offered by our health care network between April and December 2020. Chi-square tests of association were used to describe the characteristics of and differences between groups. Logistic regression analysis was used to determine significant predictors of enrollment and completion.

**Results:**

Those who enrolled in the VC platform were significantly likelier to be younger and college educated than those who enrolled in the CC platform. Those who completed a program, regardless of delivery mode, were likelier to be older and college educated than those who did not complete a program. Multivariate analyses indicated that of those enrolled in the CC platform, completers were likelier to not be enrolled in Medicaid. Among those enrolled in the VC platform, completers were older, college graduates, female, and likelier to have reported poorer health than those who did not complete the program.

**Conclusions:**

The transition of self-management programs to remote delivery modes, particularly to those that rely on VC platforms, revealed that certain demographic groups may no longer be able or willing to access the service. Efforts need to be made to increase engagement in remote self-management workshops. In addition, equivalent quality services that do not rely on a digital platform must continue to be offered in order to promote health equity.

## Introduction

The COVID-19 pandemic has resulted in skyrocketing utilization of digital platforms for health-related services, such as provider visits, support groups, and wellness classes [1-3]. Digital platforms provided a safer alternative to in-person meetings during the peak of the COVID-19 pandemic, and in some cases, improved access by facilitating more timely appointments or eliminating the need to travel [4-7]. However, in the rush to implement remote health-related services, we inadvertently risk increasing inequities to accessing care when we fail to consider the consumer groups who are unable or unwilling to use remote modes of delivery. When chronic disease self-management education (CDSME) programs switched to remote delivery modes in 2020, our team had the opportunity to explore how the delivery mechanism of the program affected participant engagement.

The evidence-based suite of chronic disease self-management programs, originally developed at Stanford University and now licensed through the Self-Management Resource Center, is a nationally disseminated community-based intervention. The program process and content are based on Bandura’s [8] social learning theory and focus on improving self-efficacy so that people are better able to manage their chronic conditions.

Typically, 2 trained peer leaders facilitate the small group workshops, which consist of 6 weekly 2.5-hour sessions. Even though leaders follow a scripted curriculum, the programs are designed to be highly participatory with group participants tailoring the content through brainstorming, pairing and sharing, and problem-solving activities (eg, if a participant states that he/she has had trouble communicating effectively with a health care provider and would like some ideas, the group will embark on a structured problem solve). In the United States, workshop delivery is usually funded by community-based organizations (eg, area agency on aging and senior center) or governmental agency (eg, local health department) and, thus, are generally offered at no cost to the participant.

The suite of CDSME workshops, which includes the Chronic Disease Self-Management Program, the Diabetes Self-Management Program, and the Chronic Pain Self-Management Program among others, has demonstrated effectiveness at improving a variety of health-related outcomes such as increased medication adherence, decreased depression, improved diabetes self-efficacy, improved pain self-efficacy, better communication with the health care team, and a reduction in hemoglobin A1c levels [9-13]. Additionally, participation in the program may reduce overall cost of care for those with chronic conditions [14,15].

The in-person workshops have been shown to be effective for individuals from a variety of backgrounds [11,16-21] including rural and minority populations and those with lower socioeconomic status (SES). However, recruitment of these populations is often challenging [22-24] owing to barriers associated with transportation, work and family obligations, and cultural beliefs [18,25,26].

Prior to the COVID-19 pandemic, our health care system had offered the Chronic Disease Self-Management Program, the Diabetes Self-Management Program, and the Chronic Pain Self-Management Program in small, in-person groups. In response to the pandemic, the Self-Management Resource Center developed two additional delivery modes: digital “in-person” sessions via videoconference (VC) using a web-based platform and a mailed toolkit with weekly small group telephone conference calls (CC). The VC format is similar in time and attention to the in-person program—that is, a 2.5-hour meeting once a week for 6 weeks with 2 peer leaders facilitating the workshop. The small group CC with a mailed toolkit is facilitated by a peer leader for 30-60 minutes per week for 6 weeks with the expectation that participants will review tool kit resources independently.

This analysis has 2 purposes. First, we wanted to ascertain enrollment and completion characteristics among the different remote delivery modes of CDSME workshops offered in our health care network’s region during the first year of the COVID-19 pandemic. Second, we wanted to determine the individual level characteristics that drove enrollment and completion across the different delivery modes. The findings from these analyses may identify groups that are at risk for inequitable access to remote health education services during and after the COVID-19 pandemic. An evidence-based program that does not reach the target population or has high rates of attrition jeopardizes the overall impact of the intervention and can potentially exacerbate existing health disparities.

## Methods

### Settings and Participants

The remotely delivered workshops were offered throughout an 8-county region in upstate New York. The region is largely rural, with an average population density of 55 residents per square mile (ie, 21 residents/km^2^), compared to 238 residents per square mile (ie, 92 residents/km^2^) in New York, excluding New York City [27]. Similar to other rural areas in the northeast, the population is predominantly White, non-Hispanic. Nearly 30% of the general population is at least 60 years old.

Participants who enrolled in 1 of 2 workshop delivery modes (VC or CC) between April and December 2020 and consented to have their data deidentified and shared for research purposes are included in this analysis. To be considered “enrolled,” an individual had to complete the baseline survey and register and receive materials for the workshop.

### Ethical Considerations

The implementation, delivery, and evaluation of the CDSME workshops within our health care system’s service region was originally developed as a quality improvement project and was determined to be exempt from ongoing oversight by the Mary Imogene Bassett Hospital's institutional review board. Participants provided written consent to attend the workshop and to have their deidentified data collected for research and evaluation purposes. Participants received a US $30 gift card to a local grocery store as compensation for completing data collection activities. Individuals who did not consent to have their data collected and hence not included in the analyses, were still able to participate in the workshop.

### Measures

Sociodemographic data (age, gender, education, marriage status, and insurance status) were collected either through paper or electronic questionnaires at baseline. In addition to sociodemographic questions, participants were asked to complete a single-item, self-rated health question [28]. All variables were dichotomized after data collection. Process measures included enrollment information (including workshop delivery mode), attendance, completion status, and reasons for not being able to attend all sessions of the workshop.

### Statistical Analyses

The analyses described below were used to answer the following questions: what are the characteristics of participants enrolled in each mode of remote workshop? What are the differences between completers and noncompleters across workshop delivery modes? What are the differences between completers and noncompleters within each delivery mode?

Descriptive statistics were used to characterize the entire sample, as well as for enrollment in each delivery mode. Bivariate analyses were conducted using chi-square tests of association to determine which characteristics were significant between groups for enrollment, an overall comparison of completers versus noncompleters, and which factors influence the likelihood of completion within each delivery mode. Based on these findings, multivariate analyses using logistic regression were conducted to explore which characteristics were independent predictors of enrolling in or completing a particular delivery mode. In all analyses, noncompleters included individuals who failed to show up for the workshop (eg, “no shows”) as well as those who showed up for fewer than 4 sessions.

Data were analyzed with SPSS (version 27; IBM Corp). Four outliers were identified in SPSS and were removed from the data set prior to analyses.

## Results

### Enrollment

Between April and December 2020, a total of 183 individuals who consented to share their deidentified data enrolled in a VC or small group CC self-management program workshop (2 additional individuals enrolled in a workshop but did not consent to share their data). The majority of the participants across workshops were older (mean age 58.6 years, median 60.0 years), female (n=143, 78.1%), married or partnered (n=93, 52.5%), and self-rated their health as good or better (n=155, 85.6%). Participant demographic characteristics are summarized in Table 1. There were significant differences in age and education level among users of the 2 different delivery modes at enrollment. Those who enrolled in the VC workshop were 3.44 times more likely to be aged 60 years or younger and 2.24 times more likely to be a college graduate (Table 1). Gender, marriage status, self-reported health, and Medicaid status were not significant indicators of program mode enrollment (*P*=.19, .76, .45, and .11, respectively).

In the multivariate analysis, age and education remained significant predictors of enrollment. The logistic regression model was significant overall (*χ*^2^_6_=22.3, *P*<.001). Those who were 60 years old or younger were significantly likelier to enroll in VC than those older than 60 years (odds ratio [OR] 3.16, 95% CI 6.26; *P*<.001). College graduates were also significantly likelier to enroll in VC than those without college education (OR 2.02, 95% CI 1.00-4.09; *P*=.05).

**Table 1:** Demographic characteristics of the sample (N=183) by program delivery mode (videoconference [VC] or conference call [CC]) and differences between groups.

Characteristics		Total, n (%)	VC (n=99), n (%)	CC (n=84), n (%)	Chi-square (*df*)		Odds ratio (95% CI)		*P* value	
**Age (years)**						16.3 (1)		3.44 (1.87-6.33)		<.001
	≤60	95 (51.9)	65 (65.7)	30 (35.7)						
	>60	88 (48.1)	34 (34.3)	54 (64.3)						
**Gender**						1.8 (1)		1.64 (0.79-3.43)		.19
	Male	38 (21)	24 (24.7)	14 (16.7)						
	Female	143 (79)	73 (75.3)	70 (83.3)						
**Marital status**						0.1 (1)		1.10 (0.61-1.99)		.76
	Married	93 (52.5)	52 (53.6)	41 (51.2)						
	Not married	84 (47.5)	45 (46.4)	39 (48.8)						
**Education level**						6.4 (1)		2.24 (1.19-4.19)		.01
	College graduate	67 (37.2)	45 (33.3)	22 (27.2)						
	Less than college education	113 (62.8)	54 (66.7)	59 (72.8)						
**Self-reported health status**						0.6 (1)		1.39 (0.59-3.25)		.45
	Fair or poor health	26 (14.4)	16 (16.2)	10 (12.2)						
	At least good health	155 (85.6)	83 (83.8)	72 (87.8)						
**Medicaid status**						2.7 (1)		0.58 (0.30-1.12)		.11
	Medicaid	53 (30.3)	62 (64.6)	60 (75.9)						
	No Medicaid	122 (69.7)	34 (35.4)	19 (24.1)						

### Completion

There were significant differences between program completers and noncompleters in terms of age, education, and self-reported health (Table 2). Completers likelier to be older than 60 years (OR 2.76, 95% CI 1.51-5.02), have a college education (OR 2.50, 95% CI 1.34-4.68), and report poorer general health (OR 4.60, 95% CI 1.65-12.81) than noncompleters.

These differences remained significant in the multivariate analyses. The logistic regression model was significant overall (*χ*^2^_6_=22.5, *P*<.001). Workshop completers were likelier be older than 60 years (OR 3.10, 95% CI 1.53-6.31; *P*=.002), be college graduates (OR 2.52, 95% CI 1.22-5.21; *P*=.01), and report poorer health (OR 4.26, CI 1.30-13.99; *P*=.02). In addition, gender emerged as a significant predictor, with females being likelier to complete a workshop than males (OR 2.40, 95% CI 1.03-5.62; *P*=.04).

When the data were stratified by workshop type (CC and VC), the CC logistic regression model (*χ*^2^_6_=16.7, *P*=.01) revealed that Medicaid status was the only independent predictor of completing a CC workshop. Those not enrolled in Medicaid were likelier to complete the program than those enrolled in Medicaid (OR 4.17, 95% CI 1.16-15.07). For the VC logistic regression model (*χ*^2^_6_=24.8, *P*<.001), age, gender, education, and self-reported health remained independent predictors of program completion, in that, those who were older than 60 years (OR 5.56, 95% CI 1.81-17.10), female (OR 4.24, 95% CI 1.16-15.43), college graduates (OR 2.77, 95% CI 1.01-7.56), and those who reported poor or fair health (OR 7.00, 95% CI 1.44, 34.07) were likelier to complete the workshop (Table 3).

**Table 2:** Differences between program completers and noncompleters.

Characteristics			Completers (n=95), n (%)		Noncompleters (n=88), n (%)		Chi-square (*df*)			Odds ratio (95% CI)			*P* value	
**Age (years)**								11.2 (1)			2.76 (1.51-5.02)			<.001
	>60	57 (60.0)		31 (35.2)										
	≤60	38 (40.0)		57 (64.8)										
**Gender**								1.2 (1)			0.71 (0.34-1.45)			.28
	Male	17 (17.9)		21 (24.4)										
	Female	78 (82.1)		65 (75.6)										
**Marital status**								0.6 (1)			1.27 (0.70-2.29)			.43
	Married	52 (55.3)		41 (49.4)										
	Not married	42 (44.7)		42 (50.6)										
**Education level**								8.4 (1)			2.50 (1.34-4.68)			.004
	College graduate	44 (47.3)		23 (26.4)										
	Less than college education	49 (52.7)		64 (73.6)										
**Health status**								9.7 (1)			4.60 (1.65-12.81)			.002
	Fair or poor health	21 (22.1)		5 (5.8)										
	At least good health	74 (77.9)		81 (94.2)										
**Medicaid status**								3.4 (1)			1.83 (0.95-3.53)			.07
	No Medicaid	69 (75.8)		53 (63.1)										
	Medicaid	22 (24.2)		31 (36.9)										

**Table 3:** Logistic regression analysis for predictors of completion split by delivery mode.

Predictors		*B* (SE)	Wald chi-square test (df)	*P* value	Odds ratio (95% CI)
**Workshop delivery mode: conference call**					
	Age>60 years	0.617 (0.563)	1.202 (1)	.27	1.85 (0.62-5.59)
	Female gender	0.271 (0.673)	0.162 (1)	.69	1.31 (0.35-4.90)
	Not married or partnered	0.415 (0.570)	0.530 (1)	.47	1.52 (0.50-4.63)
	College graduate	1.258 (0.672)	3.446 (1)	.06	3.48 (0.93-13.00)
	Self-reported less than good health	1.484 (1.137)	1.703 (1)	.19	4.41 (0.48-41.00)
	Not enrolled in Medicaid	1.429 (0.655)	4.754 (1)	.03	4.17 (1.16-15.07)
**Workshop delivery mode: videoconference**					
	Age>60 years	1.716 (0.573)	8.959 (1)	.003	5.56 (1.81-17.10)
	Female gender	1.443 (0.659)	4.791 (1)	.03	4.24 (1.16-15.43)
	Not married or partnered	–0.537 (0.521)	1.066 (1)	.30	0.58 (0.21-1.62)
	College graduate	1.019 (0.515)	3.920 (1)	.048	2.77 (1.01-7.59)
	Self-reported less than good health	1.945 (0.808)	5.700 (1)	.02	6.99 (1.44-34.06)
	Not enrolled in Medicaid	–0.538 (0.551)	0.953 (1)	.33	0.58 (0.20-1.72)

## Discussion

### Principal Findings

The purpose of these analyses was to gain a better understanding of the characteristics of those who enroll in and complete novel remote delivery modes of CDSME workshops that were developed in response to the onset of the COVID-19 pandemic. Our findings suggest that younger individuals were likelier to enroll in the VC platform than older individuals. However, older individuals were likelier to successfully complete any self-management workshop, regardless of delivery mode. When controlling for all variables within the multivariate models, not being enrolled in Medicaid significantly predicted CC workshop completion, while being older, female, a college graduate and rating one’s health as fair or poor were all independent predictors for completing a VC workshop.

The lower rate of digital technology use among older populations has been well documented [29], and the disparity has been highlighted during the COVID-19 pandemic [30,31]. However, of those who did enroll, those who were older than 60 years were likelier to complete the program, suggesting that once engaged with the remote delivery platform, older adults are able and willing to complete the workshops. Other researchers have demonstrated that self-management workshop completion rates are much higher among older cohorts [20]. Thus, while the VC delivery mode may initially engage those who are younger, we found it difficult to retain younger individuals in this program. A possible explanation for this finding is that younger and middle-aged adults are more likely engaged in the workforce and with family obligations and are hence unable to commit to a 6-week program, even if the program is offered in the evening [7,32]. This could have been particularly true during the first months of the pandemic when many parents suddenly found themselves essentially home-schooling their children. Another possible reason is that participants may be likelier to drop out if they have difficulty identifying with others in the group (eg, most participants are female, older, and retired) [33,34].

Self-management programs have typically struggled with recruiting and retaining men [35]. Our analyses are in agreement with nationwide studies, in that, nearly 80% of the participants were female. Women were also likelier to complete the program overall. However, it is interesting to note that there was no difference in completion rates between genders for the CC delivery mode. A possible explanation is that the CC mode allows for some level of anonymity; therefore, men may not feel they have to conform as much to masculine help-seeking behaviors. The CC group size is also smaller, and fewer participants per session have been shown to increase retention rates among men enrolled in the self-management programs [35].

The analyses revealed that education level was a significant factor for enrolling in and completing the VC workshop. This finding raises questions about the utility of the VC workshop for individuals who do not have a college education, particularly if one of the delivery modes demonstrates superior health-related outcomes in future randomized controlled trials. The availability of a non–internet-based remote option for those who either did not have internet access or who prefer not to use the internet is important from a health equity standpoint. However, at this time, it is unclear if the CC delivery mode has similar outcomes as the VC platform, particularly since it is shorter in time and attention.

Education level and Medicaid status are often used as proxies for socioeconomic status (SES) [36]. The digital divide is well documented with those of lower SES having less access to and lower usage of web-based services [37,38]; therefore, the abovementioned finding is not surprising. While a nationwide evaluation of the in-person CDSME workshops did not examine differences in education between completers and noncompleters, a Canadian study by McGowan found that education level was not a significant factor for completing in-person self-management workshops [13]. Our in-person workshop findings also indicated no significant difference in education level between completers and noncompleters [39,40].

A mixed methods investigation on access to, use of, and benefits from digital health services also found that being older and having less education affected access to digital health services, as well as living in a lower-SES area. Having less education was also associated with less use of digital health services [41]. However, the largest factors that influenced use were trust in digital health services, eHealth literacy, and confidence in using them. While further research is needed to understand the determinants of trusting digital health services, it is clear that a trusted provider’s recommendation to utilize the service may increase the level of trust in digital services, especially among underserved populations [41].

In this analysis, not being enrolled in Medicaid predicted completion of the program through the CC delivery mode, but did not predict program completion through the VC delivery mode or enrollment through either program delivery mode, which was an unexpected finding. Recently, investigators have attempted to understand the relationship between SES and engagement in self-management programs. A review by Hardman et al [24] on the moderating effect of SES on self-management interventions found that there was some influence of SES on attrition rates, but the lack of high-quality research made it difficult to draw conclusions. Hardman et al [24] recognized the heterogeneity of low-SES groups, and that appropriate interventions or recruitment methods depend largely on the context of that group.

A scoping review of scoping reviews also illustrated the complexities of engaging individuals in web-based health services, recognizing how an individual’s culture and perceived effectiveness of telehealth technologies is intertwined not only with digital literacy but also the social and structural determinants of health [42]. It is likely that there are other factors that we did not measure, which are stronger predictors of completion of the VC delivery mode (eg, digital literacy and trust in technology). From a health equity standpoint, we recommend further research to explore the complex relationships among education, insurance status, SES, the social and structural determinants of health, and remote workshop completion status.

Finally, it is important to recognize the effect that self-reported health status may have on the odds of workshop completion. The overwhelming majority of participants rated their health as good or better; nonetheless, over 80% of those reporting fair or poor health completed the program, compared to 47% of those reporting good or better health. While our sample size was small, leading to very large CIs, this finding does illustrate 2 important points. First, it was difficult to recruit those who rate their health as fair or poor. This finding is in line with that of other research indicating that individuals with poorer self-reported health perceive fewer benefits from digital health services [43]. However, our data show that once these individuals are engaged, they are likely to complete the program. Therefore, exploring ways to engage those who are facing poor health is critical. To do this, program implementers must acknowledge and account for the many social determinants of health, including cultural and environmental determinants, which can impact health outcomes [24,41,43,44].

### Limitations

There are several limitations that must be acknowledged in this analysis. First, the analysis was a service evaluation and was not based on an experimental design. Therefore, it is impossible to determine any causal relationships. Second, external constraints on program delivery dictated how often each mode of workshop was offered, which could have added additional bias into our findings. We also had a very small sample of individuals who reported less than good health, which may affect the accuracy of those findings. In addition, our sample population was from one particular region of New York, and the results are not generalizable to other areas. Finally, the evaluation took place during the first 10 months of the pandemic, which likely influenced health-seeking behaviors. As individuals have settled into the peripandemic era, remote workshop usage patterns may have shifted.

### Conclusions

Despite these limitations, our analysis demonstrates strong signals that certain demographic groups are less likely to initially engage with web-based CDSME workshops, particularly those who are older and less educated. In addition, completion of web-based workshops is likelier for those who have a college education, as well as those who are older and female. The traditional CC workshop appeared more accessible to participants. As health care resources rapidly become more digitized, health educators and practitioners must remain aware of this potential disparity, and future work needs to focus on understanding the nuances underlying these disparities and how we can effectively work with our patients and communities to overcome them. Remote program offerings, including those that are independent or asynchronous, can remove barriers that prevent individuals with chronic disease from engaging with in-person self-management workshops, such as transportation issues, childcare concerns, fatigue, and pain [7,45]. However, equivalent, high-quality service that does not rely on a digital platform must continue to be offered to promote health care equity.

## Abbreviations

CC: conference call
CDSME: chronic disease self-management education
OR: odds ratio
SES: socioeconomic status
VC: videoconference

## Data Availability

The data sets generated during or analyzed during this study are available from the corresponding author on reasonable request.
